# Therapeutic and Preventive Efficacy of an Intervention on Workers in a Back School

**DOI:** 10.3390/ijerph19021000

**Published:** 2022-01-17

**Authors:** Alberto Benito Rodríguez, Hugo Guillermo Ternavasio-de la Vega, José Ángel Santos Sánchez, Helena Iglesias de Sena, Miguel Marcos, Antonio Javier Chamorro, José Antonio Mirón-Canelo

**Affiliations:** 1 Engineering and Expert in Universal Accessibility, Polytechnic School of Zamora, Department of Construction and Agronomy, University of Salamanca, 37008 Salamanca, Spain; albero@usal.es; 2 Internal Medicine, Virgen de la Concha Hospital of Zamora (SACYL), 4900004 Zamora, Spain; guillermoternavasio@gmail.com; 3 Department of Biomedical and Diagnostic Sciences, Faculty of Medicine, University of Salamanca, 37008 Salamanca, Spain; jasalao@usal.es (J.Á.S.S.); hidesena@usal.es (H.I.d.S.); 4 Internal Medicine, Faculty of Medicine, University Hospital of Salamanca (SACYL), 37007 Salamanca, Spain; mmarcos@usal.es; 5 Institute of Biomedical Research of Salamanca (IBSAL), 37007 Salamanca, Spain; 6 Faculty of Medicine, Adjunct of Internal Medicine, University Hospital of Salamanca (SACYL), 37007 Salamanca, Spain; 7 Preventive Medicine and Public Health, Department of Biomedical and Diagnostic Sciences, Faculty of Medicine, University of Salamanca, 37007 Salamanca, Spain

**Keywords:** dorsolumbar pathology, pain and disability, work absenteeism and disability, intervention study with follow up, back school

## Abstract

Back pain and its ailments are the main cause of absenteeism and sick leave. Furthermore, the cause of pain and disability in a large number of workers is unknown, and treatments are not effective in controlling it. For this reason, the Back Schools (BSs) provide theoretical and practical training to workers so that they can acquire knowledge and skills that will allow them to adequately manage their back problems, enabling them to recover their autonomy and prevent relapses. The aim of the study is to analyse the efficacy of a BS by means of the evaluation of pain and disability scales in workers in different sectors and in construction. The most important clinical benefits obtained after the intervention of a BS are the reduction of pain and disability. Statistically significant and clinically relevant results have been observed between the initial assessment and the 6-month review. BS has been shown to be effective in reducing low back and neck pain and disability during the first 6 months of follow-up. Construction workers have pain and disability rates at the overall mean and with improvements between the initial assessment and the 6-month review. Their rates of improvement are clinically more relevant than for the overall population analysed.

## 1. Introduction

Spine pathology is the most expensive industrial disease, the main cause of disability in individuals under 45 years of age and the main reason for medical consultation, both in primary care and in hospitals. Back pain is one of the oldest and most frequent ailments of humanity, probably arising as a consequence of the development of bipedal ambulation and the requirement for a flexible spine [1]. Approximately 85% of cases of dorsolumbar pain have no known cause and most treatments have not been effective for its control; in fact, it has come to be considered one of the greatest failures of present-day medicine, since chronic pain has not been solved and disability has not been reduced.

Currently, dorsolumbar pain is a problem that affects 70–80% of the general population at some point in their lives, of which approximately 15% cases have a clear origin; the rest is considered non-specific or unclassifiable. At the origin of this pain are biological, psychological, social and behavioural factors and other processes that influence its chronification. It is the main cause of incapacity and absenteeism from work, with consequent high economic cost and deterioration in the quality of life of those who suffer from it [2,3].

Spine pathology is currently the most expensive industrial disease and the main cause of disability in individuals under 45 years of age [4]. The cost generated by transient disability of dorsolumbar origin is higher in Spain than in neighbouring countries of the European Union [5]. In European countries, it represents a cost of between 1.7% and 2.1% of the Gross Domestic Product (GDP), which represents a significant economic expenditure and consumption of health resources [3]. At present, dorsolumbar pathologies are the main cause of absenteeism and sick leave in Spain, representing a very important socioeconomic cost and a major public health problem due to their high prevalence and social and health repercussions, generating demand for consultations, high use of health services and, above all, a considerable loss of working days. It is considered the main reason for medical consultations relating to the locomotor system, both in primary care and in hospitals, as between 60–90% of the population will suffer an episode of low back pain at some point in their lives [6].

Its prevention in the labour environment is fundamental, since avoiding its appearance can reduce the incidence of musculoskeletal disorders (MD) and reduce its economic impact in the workplace. However, the traditional way of managing and treating low back pain, using the “anatomoradiological model”, has come to be considered one of the greatest failures of medicine, due to the fact that it has not managed to resolve the aetiopathogenesis and that therapeutic means have not solved chronic pain or reduced disability [7]. Other authors support this hypothesis by stating that low back pain is an important health problem that has not been resolved by the usual treatments [8]. Given the failure of empirical treatments, the chronification of a significant number of processes and the large number of incapacities, it is suggested that patients should deal with their pain by means of good information and exercises aiming at preventing and treating their ailment [9].

In this health and work context, the Back School (BSs) appear as a method to educate the patient, evaluating the possible causes and mechanisms of low back pain, giving advice on postural hygiene and recommending back exercises, depending on the needs of the patients [10]. Therefore, the BS becomes a therapeutic alternative for both primary and secondary prevention of chronic low back pain that aims to make the patients aware of their pathology, so that they can acquire healthy habits and get involved in its management and self-care [1]. The BSs have shown themselves to be an effective tool for therapeutic intervention, as it has been demonstrated that Back School programmes are effective in the treatment of non-specific low back pain [11].Their most important benefits are the reduction of pain and the decrease of disability. In addition, it has been shown that low-cost exercise programmes can provide enormous relief in therapeutic processes, rehabilitation therapies and, therefore, cost savings for the health care system [11,12].

The general objective of this work is to analyse the effectiveness of BSs in the workplace and specifically in the construction sector, analysing risk factors and the scale of pain and disability.

## 2. Materials and Methods

From the scientific approach, statistically, the hypotheses are as follows:

– Null Hypothesis (Ho): Attendance at the Ibermutua BS does not have a favourable and positive influence on the evolution of the indicators of pain and incapacity of workers.
– Alternative Hypothesis (Hx): Attendance at the Ibermutua BS has a favourable and positive influence on the evolution of the indicators of pain and disability of workers.

In addition to the hypotheses, a complementary aim is to determine the existence of an association between variables such as age, gender, acquisition of knowledge in postural hygiene, increase in physical activity, work situation and previous history, with the patient’s improvement due to BS. More specifically, the influence of work activity in a sector that is as apparently harmful to the back as construction is analysed.

### 2.1. Sample of Workers and Type of Study

The initial sample for this study drew upon an initial database of 3281 workers with low back or neck pain, who participated in the BS programme of the Mutua de Accidentes de Trabajo y Enfermedades Profesionales Ibermutua between 1 April 2009 and 28 March 2019, in Spain. The study combined observation and follow-up over a decade.

The design used to achieve the objectives and evaluate the hypotheses consisted of a descriptive, analytical and prospective multicentre intervention study of workers included in Ibermutua’s BS programme, from different sectors and trades who have participated in BS activities in 30 different Spanish provinces. A descriptive study was carried out on the 3282 workers who attended the first session and a prospective study on patients who completed the second and third BS check-ups at 6 and 12 months.

### 2.2. Programme Structure and Scales for Assessing the Outcome of the Intervention

For the evaluation and analysis of the results, the questionnaires and scales selected were as follows. Pain assessment scales: lumbar visual analogue scale (lumbar VAS) and cervical visual analogue scale (cervical VAS) [13]. Disability assessment scales: neck disability index (NDI) or neck disability questionnaire (NDQ) [13] and lumbar disability index or Oswestry disability questionnaire (ODQ) [14,15]. These tests were taken once at the initial session, and then repeated at 6 months and a final session at 9 months.

### 2.3. Epidemiological Variables Studied

The BS consists of a structure made up of 3 sessions. In the first session, the data collection questionnaire was completed, the aim of which was to collect a series of data on the patient who was undergoing the BS programme in order to establish the socio-demographic and clinical characteristics, as well as to establish exactly which patients were candidates to be included in the programme. In addition to the variables collected, a “test of understanding of concepts” was carried out on the concepts explained, consisting of a battery of 35 questions that allowed the degree of knowledge acquired by each of the patients in the BS programme to be assessed for its application in their daily lives. Each of the 35 items had two possible answers, True (T) or False (F). The level of understanding of concepts was assessed according to the following parameters: high—>90% correct answers, medium—75–90% correct answers and low—<75% correct answers. In addition to the two questionnaires, the following were also completed in this first session: Visual Analogue Scale (VAS) Lumbar and Neck, Oswestry Low Back Pain Disability Questionnaire and the Neck Disability Index. These same tests were repeated in the 2nd and 3rd session at the 6 and 9-month check-ups, respectively.

The demographic and clinical variables were as follows:

Demographic: Sex, age, place of residence, sector of work activity, occupation and activity with standing and prolonged sitting, risk perception (high or low), work status (active, low or disability) and job satisfaction.

Clinical: Location of pain (lumbar, cervical), consumption of analgesia, level of physical activity, family history of back pain and personal history of back pain, number of episodes (prior to current/yearly) and degree of compliance and level of adherence to treatment.

### 2.4. Statistical Analysis

Statistical analysis was performed using the SPSS 26.0 package for Windows (SPSS^®^ Inc., Chicago, IL, USA), based on data entered using an EXCEL^®^ spreadsheet. A blinded data release by Ibermutua was considered to guarantee strict anonymity.

The Kolmogorov–Smirnov test was used to study the normal distribution of the variables. Each quantitative variable was described with central measures and standard deviation (SD), and each qualitative variable was described with the distribution of absolute frequencies and/or percentages. The degree of relationship between quantitative variables was determined using Pearson’s correlation coefficient (r). For intergroup comparisons between qualitative and quantitative variables, a Student’s *t*-test for independent samples was performed. To compare paired samples, a hypothesis test was performed using Student’s *t*-test for related data. The level of statistical significance was set at *p* < 0.05.

## 3. Results

### 3.1. Gender

Of the 3281 students who participated in the Ibermutua BS between 2009 and 2019, 1452 (44.25%) were women and 1829 (55.75%) were men. The age range coincided for both sexes and was between 22 and 77 years old. As can be seen from the results in Table 1 obtained during the first BS session, a higher proportion of pain and disability was observed in women than in men, with statistically significant differences.

Regarding the construction sector, out of the total of 316 workers participating in the BS, 297 were men and only 19 women, or 6%. For this reason, the gender aspects of this field of work have not been contrasted.

### 3.2. Age

Ages ranged from 22 to 77 years, with an average age of 50.28 years and a standard deviation (SD) of 10.08. The mean age was used to establish two groups with ≥50 and ≤50 years. Table 2 shows the means of low back and neck pain, as well as the percentages of incapacity reported by the workers.

When applying the Pearson correlation coefficient contrast, it is observed that those aged ≥50 suffer greater low back pain (VAS with *p* = 0.02) and disability (lumbar and cervical) with statistically significant differences (*p* = 0.0012 and *p =* 0.016, respectively).The mean age of construction workers was 50.67 years (9.7 SD) with an age range between 30 and 72 years. The statistical analysis between pain and disability (lumbar and cervical) of construction workers according to age, showed similar results to the rest of the workers (see Table 3).

In the construction sector, the data obtained using Pearson’s correlation coefficient are statistically significant in the degree of lumbar disability (*p =* 0.001), but not in cervical disability and in the assessment of pain.

### 3.3. Purpose of BS

The students undertook BS for both therapeutic and preventive purposes. According to the data, 519 (16.1%) did BS for preventive purposes and 2539 (78.7%) for therapeutic purposes. 170 (5.2%) workers (out of a total of 3282) did not answer. Workers who undertook BS for therapeutic purposes reported more pain and disability (both lumbar and cervical) than those who undertook BS for preventive purposes, with statistically significant differences. The summarised data can be seen in the following table (see Table 4):

The statistical analysis of the pain and disability averages of construction sector workers according to the purpose of the BS yielded similar results to the rest of the workers as shown in Table 5.

### 3.4. Developments in BS Monitoring

A total of 8760 tests were performed to measure pain and disability, distributed as follows: Initial session: 7395 (5067 lumbar and 2328 cervical); 6-month review: 752 (401 lumbar and 351 cervical); 9-month review: 613 (330 lumbar and 283 cervical). On analysing the evolution of each of the questionnaires, it can be seen that there is a large drop in the number of questionnaires completed between the 1st and 2nd session, and that this drop stabilises between the 2nd and 3rd session.

**Figure 1:**
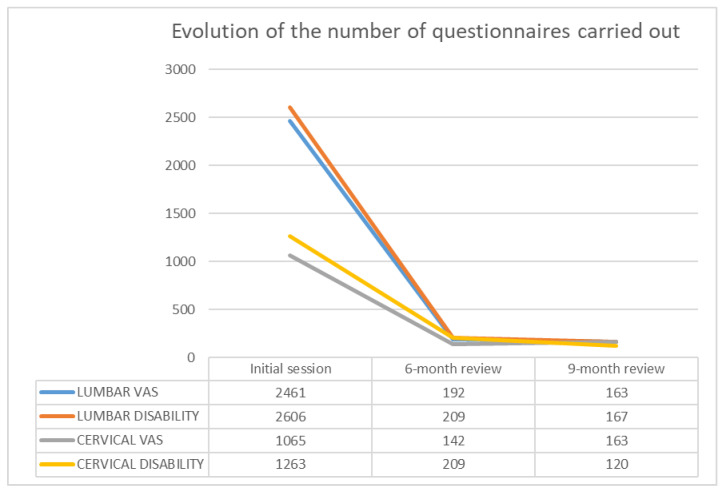
Time evolution in the number of questionnaires, in the 1st evaluation of the BS, 2nd review at 6 months and 3rd review at 9 months.

Analysing the tests by gender, the following results were obtained (see Table 6):

The parallelism between the lumbar and cervical pain and disability tests could also be appreciated. The following figure shows the observations obtained (see Figure 1).

Then, we analysed whether pain or degree of disability influenced DTS follow-up (see Table 7).

### 3.5. Understanding of the Theoretical-Practical Content of the Back School

At the end of the BS programme, a questionnaire was used to measure the degree of knowledge acquired by students, for subsequent application in their daily lives. Regarding the level of understanding of concepts, the following correction ratios were selected: high—>90% of correct answers, medium between 75–90% and low ≤75%. The results obtained by the students were 73% high, 24% medium and 4% low. Only 3% of the total number of students who undertook the concept comprehension questionnaire (n = 2382) obtained a correct rate of less than 75% (low level of comprehension).

### 3.6. Clinical Situation

Of the 3282 workers included in the BS, 2598 (67.3%) reported some type of pathology, pain or discomfort in the lumbar area and 1264 (32.7%) in the cervical area.

The total number of questionnaires completed on the low back, lumbar VAS and Oswestry disability (5059) was significantly higher than those completed on the neck VAS and neck disability questionnaires (2330).

A total of 8750 tests were performed to measure pain and disability, distributed as follows. Initial session: 7385 (5059 from the lower back and 2326 on the cervical). Review at 6 months: 752 (401 from the lumbar area and 351 on the cervical). Review at 9 months: 613 (330 from the lumbar area and 283 on the cervical).

In addition, 1800 data collection questionnaires and 2376 concept comprehension assessments were completed. The data obtained from the questionnaires completed in the first session of the BS were distributed as follows. Lumbar VAS: 2461 and Cervical VAS: 1067 questionnaires. Oswestry disability: 2597 questionnaires. Cervical disability: 1260 questionnaires.

### 3.7. Effectiveness of VAS on Low Back Pain

**Figure 2:**
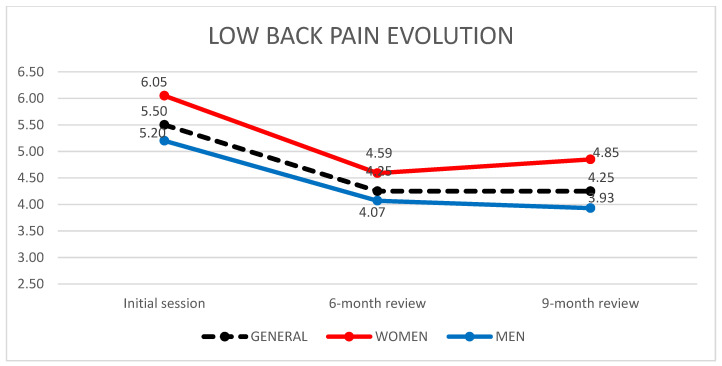
Evolution of the average low back pain, according to gender, from the workers’ VAS, carried out in the first session, review at 6 months and review at 9 months.

A total of 110 workers completed all the low back pain assessments, performing the VAS at the first session, the review at 6 months and finally the review at 9 months. Their gender distribution was 71 men (65.5%) and 39 women (35.5%). In all sessions, women reported pain averages above the general average for men (see Figure 2). Table 8 shows the evolution of lumbar VAS averages over the 3 sessions.

The statistical analysis establishes a significant decrease in VAS between the initial session and the 6-month review with *p* < 0.0001. This was not observed in the 9-month review.

**Figure 3:**
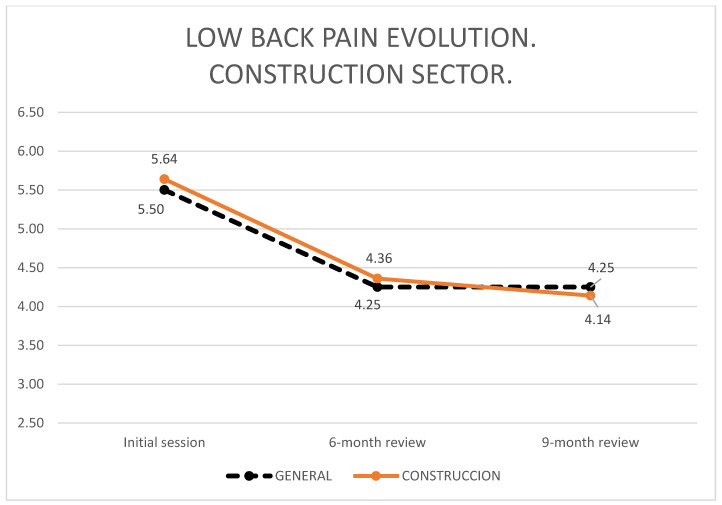
Questionnaires administered in the first BS session.

Figure 3 shows the evolution of the degree of low back pain in the in the construction sector workers who underwent all the VAS, compared with the total.

At 6 months, 77.4% of the workers in this study had no or less pain than at the start of the BS. At 9 months, the percentage decreased to 70.8%. For construction workers, the corresponding figures of workers with less or no pain were 69.2% at 6 months and 73.9% at 9 months.

34% of workers in this study claimed to have suffered relapses or new painful episodes 6 months after their time in our BS and 37% after 9 months. The percentages for workers in the construction sector were 30.8% at 6 months and 34.8% at 9 months. The ncrease in relapses at nine months suggests a lack of continuity in practice of the recommendations of the Back School by the workers. The percentage of workers who reported being on sick leave was 13.7% at 6 months and 13.8% at 9 months. In the case of workers in the construction sector, the percentage of sick leave stood at 23.1% at 6 months and 17.4% at the end of the 9-month follow-up. 13.8% of workers in this job reported having had new sick leaves in the 9 months after the start of the BS. For workers in the construction sector this figure was 8.7%.

In terms of satisfaction after completing the programme, 94.9% of the participants in the Ibermutua BS considered it to be useful. For workers in the construction sector, the satisfaction rate was 95.7%.

## 4. Discussion

The study of the Ibermutua BS population is larger than for other national BS, such as that of the Hospital General Universitario Morales Meseguer (HGUMM) with 378 patients analysed [6], 319 in a previous Ibermutua study, in the period 2006–2009 [12], or the 192 participants in the Rickets Programme of the Hospital de San Juan [16].

The population in this study shows a slight predominance of males (56%) compared to females (44%). In the BS of the HGUMM, the parameters are just the opposite, with a predominance of women (56%) compared to men (44%) [6]. In the international literature, unlike this study, BSs also show a predominance of the female sex [17]. When analysing the relationship between gender of the BS participants and pain reported by them in the initial assessment, women report greater pain and a greater degree of lumbar and cervical disability compared to men, with a statistically significant difference. Other authors also reaffirm the idea of greater pain intensity in women than in men [18]. Observational studies carried out in the workplace, which consider the different socio-occupational factors involved, also note a significant differentiation in relation to gender, with more frequent and greater numbers of painful points and pain intensity in women [19]. This is a true reflection of the reality of a sector that is highly conditioned by gender in certain work activities; in fact, the low presence of women in the construction sector makes any analysis impossible.

The average age of the participants in the Ibermutua BS was 50.28 years, 50.36 years for women and 50.22 for men. The age range was between 22 and 77 years. The average age in the construction sector was 50.67 years. In both sexes, age is associated with an increased risk of back pain. In numerous studies the prevalence of back disorders increases with age, which could be explained by the fact that the cushioning capacity of the spine decreases with time [20]. Age is therefore a non-modifiable risk factor or risk marker. In this BS, a statistically significant relationship was observed between age and lumbar and cervical disability and lumbar pain reported by the students in the first VAS; but no statistically significant relationship was found with cervical pain, despite the fact that there are studies that state that cervical pain is very frequent, particularly among the population aged ≥ 50 years [21]. In the construction sector, no statistically significant relationship has been found between pain, both lumbar and cervical, and age. There are studies along the same lines, stating that in the construction sector there is no significant relationship between musculoskeletal symptoms and age, with a higher percentage of cases found in young workers. This could be due to the fact that workers who carry out a given task learn and develop strategies over the years to perform their tasks with less risk [22]. This is a broad observation taking into account the ageing of workers in this sector [23]. As for the relationship between age and degree of disability, it can be concluded that age influences the degree of lumbar and cervical disability reported by patients ≥ 50 years of age in the Ibermutua BS, both for construction workers and for intersectoral workers. This result is consistent with the literature consulted [17,24].

Another relevant aspect is the importance of pain in occupational absenteeism: 40% of people suffering from severe pain are absent from work, compared to 3% of the population who do not suffer from pain [25]. At the start of the Ibermutua BS, 50% of workers report being on sick leave. After 6 months, the percentage drops to 14%, a figure that remains the same after 9 months. For the construction sector, the number of workers on sick leave at the initial assessment is slightly higher than the inter-sector average, at 56%, decreasing to 23% at 6 months and 17% at 9 months. In this study, statistically significant results were obtained in terms of the relationship between the pain index and the degree of disability reported in the first VAS test (both lumbar and cervical) and the work situation of discharge or sick leave, with the average pain index and disability being higher in the students on sick leave. The same analysis carried out on workers in the construction sector shows similar results, except for cervical pain, for which no statistically significant relationship is found with the worker’s sick leave status. This is probably because neck pain behaves in a special way as it also depends on psychological factors [26,27].

78.7% of the students attend the BS for therapeutic reasons and the rest for preventive purposes. In the construction sector, the percentage of workers who take the programme for therapeutic reasons is 82.6%. There is a statistically significant relationship between the purpose and the degree of pain and disability manifested, both for the construction sector and in the intersectoral analysis, with higher values being reached among workers who carry out the BS for therapeutic reasons. Sixty-seven percent of the tests performed in our BS correspond to the lumbar area and only 33% to the cervical area, in line with studies on the location of pain carried out in Spain, which determine percentages of 60.5% for the back and 28.6% for cervical pain [19]. Construction workers show even less willingness to undergo cervical tests, only 22%. In this study, it is observed that the intensity of referred pain in the lumbar region is greater than in the cervical region, with women suffering from cervical pain more frequently than men, in line with other studies consulted [19,20]. Exercise may have specific benefits in reducing the severity of chronic pain. Physical activity and exercise programmes are increasingly used and offered in various health systems. Health education and yoga are among the cost-effective and cost-efficient options for the treatment of low back pain [28,29,30,31]. In exercise, patient awareness and involvement in their recovery should be actively sought and strategies used to improve and maintain adherence, considering the patient’s preferences on the type of activity for best results [32].

In relation to the effectiveness of BS, a clinically significant decrease in low back pain was observed between the initial assessment and the 6-month review; but no improvement was observed between the 6-month review and the 9-month review.

Workers in the construction sector have mean low back pain VAS slightly above the overall mean, except at their 9-month check-up. The evolution of lumbar VAS for workers who perform all check-ups behaves in a similar way to that of workers who miss some of them, decreasing significantly from the initial assessment to the 6-month check-up, but not from the 6-month check-up to the 9-month check-up. However, it should be noted that a statistically significant change does not necessarily coincide with a clinically relevant change [33]. After reviewing the existing literature, there is an international clinical consensus to set the minimum change in significance (MIC) at 20% for the visual analogue pain scale [34]. In this study this threshold of improvement is exceeded between the initial assessment and the 6 and 9-month review, but there is no such improvement in the comparison between the 6-month review and the 9-month review. At the 9-month follow-up, an improvement of 23% is obtained, coinciding with other authors, who report the same figure of 23% in VAS over the initial assessment [12]. As in this study, other authors achieve a decrease in pain at the first check at six months without this significant difference persisting in the long term at the annual appointment [3].

In the current literature there is much variability, generally achieving a reduction in pain, although not always significantly, thus giving prominence to the reduction of disability and the improvement of some aspects of quality of life [35,36]. In fact, some schools do not focus on improving pain but rather functionality, giving greater importance to decreases in disability questionnaires than to VAS. Because pain intensity and degree of disability do not correlate, pain scales should not be used to measure disability [37]. In our opinion, it is considered positive to assess both aspects because of the strong association between them.

At the initial assessment, and considering all participating workers at baseline, regardless of whether or not they followed up, the Oswestry disability questionnaire completed by 2606 workers yields an ODQ of 32 (moderate disability). At the last review at 9 months, the ODQ drops to 18 (minimal disability). A minimum change of at least 10 points is considered reasonable to ensure that the patient has experienced an improvement [34]. The Food and Drug Administration (FDA) considers that 15 points should be the minimum relevant change in patients assessed before and after spinal fusion [38].

Of the total number of BS workers, 130 completed all the low back disability questionnaires. The initial assessment showed a 31% disability rate, 37% for women and 28% for men. Statistical analysis showed a statistically significant and clinically relevant decrease in the low back disability index between the initial session and the 6-month review, where an ODQ of 19% was achieved (an improvement of more than 12 points). Other authors report a significant improvement in the ODQ of 9% (less than that obtained in this study), starting from a baseline assessment of 27% at the initial evaluation and decreasing to 18% at 9 months [12].

Upon completion of BS, most patients report an improvement in their work situation due to the awareness and concepts of postural hygiene to apply in their daily activities. At 6 months, more than three quarters of the workers in this study report no or less pain than at the start of BS. At 9 months, the percentage drops to 70%. Similar figures are reported by construction workers, with 74% of workers having less or no pain at 9 months. At the HGUMM, most of the subjects did not report a flare-up of pain, and if they did, they were able to resolve and treat it on their own [6]. Only 34% of the workers in this study claimed to have suffered relapses or new episodes of pain 6 months after the BS and 37% after 9 months. Schools such as those in Spain also achieved a decrease in the number of crises and a shorter duration of clinical symptoms [38,39].

There is enormous heterogeneity when it comes to establishing universally accepted criteria for evaluating the outcome of treatments in patients with chronic low back pain. A wide variety of variables have been used, which makes it difficult to compare the different studies [37,38,39,40,41]. However, the main outcome measures recommended in BS studies are the assessment of pain and functional status, and it would be necessary to assess health-related quality of life as an indicator of the overall clinical and functional outcome [39,41,42,43]. A systematic review and meta-analysis on the preventive and clinical usefulness of Back Schools concluded that the evidence base used to evaluate outcomes is weak [44]. In this work, several questionnaires and scales have been used to assess the results in workers’ pain and functional status; but the authors are aware of the lack of objective indicators to measure clinical and preventive outcomes as suggested by some experts. An indicator is suggested, for example, to assess the protected lumbar flexion gain (measured in centimeters) [44].

## 5. Conclusions

First, it is observed that the more intensive the programme is, the more effective it becomes. It can be stated that the relationship between the duration of the BS and the results obtained can be considered effective and efficient.

Second, the employment situation improves significantly after the BS. The percentage of workers on sick leave is reduced from 50% at the beginning of the programme to 14% after 9 months. The results are mainly at the lumbar level and at 6 months. For construction workers, the decrease is even greater, from 57% to 17%. At 9 months, less than 14% of the workers report having had new sick leaves and 9% in the case of construction workers.

In relation to the results, there is a statistically significant and clinically relevant decrease in pain and disability, both lumbar and cervical, between the first assessment and the 6-month review, but not between the 6-month review and the 9-month review.

Third, with regard to the quality of the BS as measured by the satisfaction of the workers, once the programme has finished, 95% of the participants consider it to be useful, a figure very similar to that expressed by the workers in the construction sector. In line with other BSs, the majority of Ibermutua BS participants report high satisfaction with the programme.

## Figures and Tables

**Table 1:** First BS assessment: pain and disability by gender.

Gender	n (%)	Lumbar Stocking	SD	*p*-Value	n (%)	Cervical Stocking	SD	*p*-Value
**PAIN**								
**Female**	1.013 (41.2)	5.6	2.0	<0.001	655 (61.5)	5.3	2.1	<0.001
**Male**	1.448 (58,8)	5.2	2.1		410 (38.5)	4.6	2.3	
**DISABILITY**								
**Female**	1.061 (40.7)	35.4	17.2	<0.001	768 (39.2)	37.6	17.2	<0.001
**Male**	1.545 (50.3)	30.7	17.0		495 (60.8)	29.8	16.9	

n: sample size. Means of low back and neck pain obtained from the VAS at the initial assessment. SD: Standard Deviation. *p*-Value: statistical significance from Student’s *t*-test, difference between means for independent samples. Mean lumbar and cervical disability obtained from the Oswestry questionnaire and cervical disability at initial assessment.

**Table 2:** First assessment of BS: pain and disability as a function of age.

Age	n (%)	Lumbar Stocking	SD	*p*-Value	n (%)	Cervical Stocking	SD	*p*-Value
**PAIN**								
**(<50)**	1.195 (48.6)	5.2	2.0	0.014	536 (50.3)	5.0	2.3	0.657
**(≥50)**	1.265 (51.4)	5.4	2.1		529 (49.7)	5.1	2.2	
**DISABILITY**								
**(<50)**	1.261 (48.4)	31.5	17.0	0.002	627 (49.6)	33.5	17.4	0.019
**(≥50)**	1344 (51.6)	33.6	17.4		636 (50.4)	35.5	17.6	

n: sample size. Means of low back and neck pain obtained from the VAS at the initial assessment. SD: Standard Deviation. *p*-Value: statistical significance from Student’s *t*-test, difference between means for independent samples. Mean lumbar and cervical disability obtained from the Oswestry questionnaire and cervical disability at initial assessment.

**Table 3:** First evaluation of the BS: pain and disability as a function of age in construction workers.

Age	n (%)	Lumbar Stocking	SD	*p*-Value	n (%)	Cervical Stocking	SD	*p*-Value
**PAIN**								
**(<50)**	122 (49.6)	5.2	2.1	0.092	39 (54.2)	4.8	2.1	0.535
**(>50)**	124 (50.4)	5.6	2.0		33 (45.8)	5.1	2.7	
**DISABILITY**								
**(<50)**	133 (50.6)	29.6	16.5	0.002	41 (48.8)	28.9	14.7	0.02
**(>50)**	130 (49.4)	36.1	16.6		43 (51.2)	36.8	17.6	

n: sample size. Means of low back and neck pain obtained from the VAS at the initial assessment. SD: Standard Deviation. *p*-Value: statistical significance from Student’s *t*-test, difference between means for independent samples. Mean lumbar and cervical disability obtained from the Oswestry questionnaire and cervical disability at initial assessment.

**Table 4:** First BS evaluation: pain and disability as a function of the purpose for which the BS was performed.

Purpose	n (%)	Lumbar Stocking	SD	*p*-Value	n (%)	Cervical Stocking	SD	*p*-Value
**PAIN**								
**Therapeutic**	2.090 (85.0)	5.5	2.0	<0.001	849 (79.6)	5.3	1.2	<0.001
**Preventive**	370 (15.0)	4.3	2.2		217 (20.4)	4.2	2.3	
**DISABILITY**								
**Therapeutic**	2.189 (84.0)	34.7	16.7	<0.001	995 (78.7)	36.7	17.2	<0.001
**Preventive**	417 (16.0)	21.5	15.7		269 (21.3)	26.4	16.7	

n: sample size. Means of low back and neck pain obtained from the VAS at the initial assessment. SD: Standard Deviation. *p*-Value: statistical significance from Student’s *t*-test, difference between means for independent samples. Mean lumbar and cervical disability obtained from the Oswestry questionnaire and cervical disability at initial assessment.

**Table 5:** First evaluation of the BS: pain and disability according to the purpose of the BS for workers in the construction sector.

Purpose	n (%)	Lumbar Stocking	SD	*p*-Value	n (%)	Cervical Stocking	SD	*p*-Value
**PAIN**								
**Therapeutic**	220 (89.4)	5.5	2.0	0.003	58 (80.6)	5.3	2.3	0.010
**Preventive**	26 (10.6)	4.3	2.3		14 (19.4)	3.5	2.3	
**DISABILITY**								
**Therapeutic**	235 (89.4)	34.2%	16.7	<0.001	68 (81.0)	34.9	16.3	0.022
**Preventive**	26 (10.6)	21.2%	13.7		16 (19.0)	24.4	16.0	

n: sample size. Means of low back and neck pain obtained from the VAS at the initial assessment. SD: Standard Deviation. *p*-Value: statistical significance from Student’s *t*-test, difference between means for independent samples. Mean lumbar and cervical disability obtained from the Oswestry questionnaire and cervical disability at initial assessment.

**Table 6:** Questionnaires carried out in the first session by gender.

	Total (N)	Women (n)	(%)	Men (n)	(%)
**Lumbar VAS**	2.461	1.013	41.2	1.448	58.8
**Cervical VAS**	1.065	655	61.5	410	38.5
**Lumbar disability**	2.606	1.061	40.7	1.545	59.3
**Cervical disability**	1.263	768	60.8	495	39.2

N: study population. n: sample size.

**Table 7:** Pain and disability as a function of follow-up.

Complete All Evaluations	n (%)	Lumbar Stocking	SD	*p*-Value	n (%)	Cervical Stocking	SD	*p*-Value
**PAIN**								
**No**	2.351 (95.5)	5.3	2.0	0.426	996 (93.4)	5.1	2.2	0.229
**Yes**	110 (4.5)	5.5	2.2		70 (6.6)	4.7	2.6	
**DISABILITY**								
**No**	2.476 (95.0)	32.6	17.1	0.225				
**Yes**	130 (5.0)	31.6	18.6					

n: sample size. Means of low back and neck pain obtained from the VAS at the initial assessment. SD: Standard Deviation. *p*-Value: statistical significance from Student’s *t*-test, difference between means for independent samples. Mean lumbar and cervical disability obtained from the Oswestry questionnaire and cervical disability at initial assessment.

**Table 8:** Evolution of the mean results of the visual analogue scale for low back pain: first assessment, review at 6 and 9 months.

Complete All the Tests	LUMBAR PAIN					
	Initial Assessment		Revision 6 Months		Revision 9 Months	
	Media	SD	Media	SD	Media	SD
Total: 110	5.5	2.2	4.2	2.3	4.2	2.5
**Women**: 39 (35.5%)	6.0	2.3	4.6	2.3	4.8	2.4
**Men**: 71 (64.5%)	5.2	2.1	4.1	2.3	3.9	2.4

n: sample size. Mean lumbar disability obtained in the initial VAS and the 6- and 9-month review. SD: Standard Deviation.
